# An Insight into Geometries and Catalytic Applications of CeO_2_ from a DFT Outlook

**DOI:** 10.3390/molecules26216485

**Published:** 2021-10-27

**Authors:** Hussein A. Miran, Zainab N. Jaf, Mohammednoor Altarawneh, Zhong-Tao Jiang

**Affiliations:** 1 Department of Physics, College of Education for Pure Science, Ibn Al-Haitham, University of Baghdad, Baghdad 10071, Iraq; zainab.n.a@ihcoedu.uobaghdad.edu.iq; 2 Department of Chemical and Petroleum Engineering, United Arab Emirates University, Sheikh Khalifa Bin Zayed Street, Al-Ain 15551, United Arab Emirates; 3 Surface Analysis and Materials Engineering Research Group, College of Science, Health, Engineering and Education, Murdoch University, Murdoch, WA 6150, Australia; Z.Jiang@murdoch.edu.au

**Keywords:** cerium oxide (CeO_2_), fluorite structure, catalytic applications, density functional theory (DFT), oxygen vacancies, surface stability

## Abstract

Rare earth metal oxides (REMOs) have gained considerable attention in recent years owing to their distinctive properties and potential applications in electronic devices and catalysts. Particularly, cerium dioxide (CeO_2_), also known as ceria, has emerged as an interesting material in a wide variety of industrial, technological, and medical applications. Ceria can be synthesized with various morphologies, including rods, cubes, wires, tubes, and spheres. This comprehensive review offers valuable perceptions into the crystal structure, fundamental properties, and reaction mechanisms that govern the well-established surface-assisted reactions over ceria. The activity, selectivity, and stability of ceria, either as a stand-alone catalyst or as supports for other metals, are frequently ascribed to its strong interactions with the adsorbates and its facile redox cycle. Doping of ceria with transition metals is a common strategy to modify the characteristics and to fine-tune its reactive properties. DFT-derived chemical mechanisms are surveyed and presented in light of pertinent experimental findings. Finally, the effect of surface termination on catalysis by ceria is also highlighted.

## 1. Introduction

Rare earth (RE) elements have gained considerable interest due to the distinguished electronic configuration triggered by their 4*f* electrons. They differ from other elements in that their valance electrons can occupy more than one shell, offering them several possible oxidation states. This is clearly displayed in acquiring different electronic characteristics, spanning insulating, conducting, and superconducting behaviors [1,2]. It has been reported that oxides of rare earth elements, including Sc and Y along with lanthanoids, find a variety of applications most notably in automobile, electronic industries, catalysis, catalyst support, lighting, and biomedicine [3,4]. Along the same line of inquiry, the valence of cerium displays a significant influence on the structure of cerium dioxides in that tetravalent Ce comprises cerium dioxide (CeO_2_) that exhibits a cubic fluorite lattice (Fm$\bar{3}$m space group) and the oxygen anions occupy the eight tetrahedral 8c sites, whereas trivalent cerium displays the sesquioxide Ce_2_O_3_. The latter displays a hexagonal lattice (P$\bar{3}$m1 space group). The contribution of valence electrons to the chemical bond in CeO_2_ suggests that the Ce 4*f* electrons participate directly in chemical bond formation in CeO_2_ [5]. In addition, owing to paired and unpaired electrons in CeO_2_ orbitals, CeO_2_ shows exceptional magnetic behavior involving paramagnetism, ferromagnetism, and diamagnetism [6]. Moreover, nanostructured cerium oxide films reveal exceptional corrosion resistance performance and durability of the superhydrophobic surface [7]. It is also revealed that the oxides of rare earth metals, mainly Ce, La, and Y, could enhance the high-temperature oxidation resistance of various metal alloys up to 1000 °C [8].

## 2. Crystals Structure and Electronic Properties of Stoichiometric and Nonstoichiometric CeO_2_

**Figure 1:**
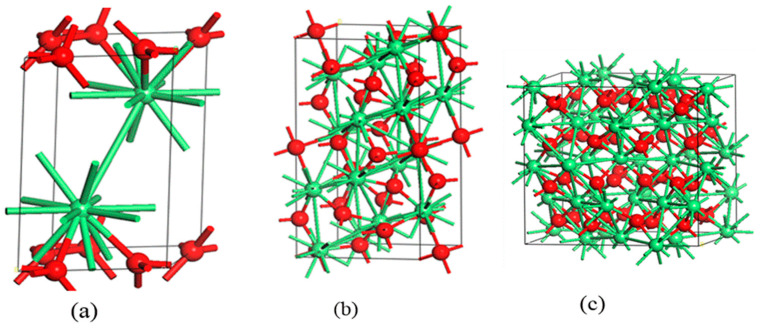
Unit cells of Ln_2_O_3_: (**a**) A-type (hexagonal), (**b**) B-type (monoclinic), and (**c**) C-type (cubic).

A great number of studies have been devoted to studying the crystal structure and geometries of RE oxides. Depending on the operational temperature and pressure, Ln_2_O_3_ species can adopt three distinct phases at temperatures below 2000 °C. The light rare earth element Ln_2_O_3_ oxides (A-type, space group of P3m1, no.164) (164 refers to Patterson space group) feature a hexagonal structure as depicted in Table 1. The heavy RE element Ln_2_O_3_ oxides (C-type, space group of Ia3, No. 206) are known to adopt cubic structures, whilst the remaining rare earth elements oxides generally crystallize in either C-type structure or B-type structure (monoclinic crystallography) [9]. All RE elements oxidize readily but to varying extents of oxidation. In the presence of air, for instance, cerium (Ce) oxidizes to ceria (CeO_2_), which possesses a fluorite structure as presented in Figure 1. On the other hand, praseodymium (Pr) occurs naturally as Pr_6_O_11_, whilst terbium (Tb) is found as Tb_4_O_7_. Both oxides transform into PrO_2_ and TbO_2_ under oxygen pressure. These three dioxides are crystallized as cubic fluorite face-centered structures. However, the rest of the lanthanide oxides are found in nature in the form of sesquioxides (Ln_2_O_3_) [10]. It is worth mentioning that most of the rare earth oxides are thermally stable and expected to be highly active against H_2_O and CO_2_. The most common oxidation state they have is that of a trivalent state, but they can also switch to either a divalent or a tetravalent oxidation state [11].

The geometric, electronic, and mechanical properties of rare earth sesquioxides and the phase transition pressure from cubic (C-type) to hexagonal (A-type) have been extensively investigated by means of first-principle calculations predominantly based on the density functional theory (DFT) approach. Richard et al. [12] calculated and validated their theoretical findings on structural and mechanical properties along with the pressure at which phase transition from cubic to hexagonal occurs against corresponding experimental values. Besides, a computational study has shed light on structural, electronic, and thermal properties of bulk and surface terbium dioxide (TbO_2_) as a material that offers wide spectra of catalytic and optical applications. The calculated lattice parameter of 5.36 Å matches well with the analogous experimental value of 5.22 Å. The density of states (DOS) of the bulk structure displays a semiconducting character [13].

**Figure 2:**
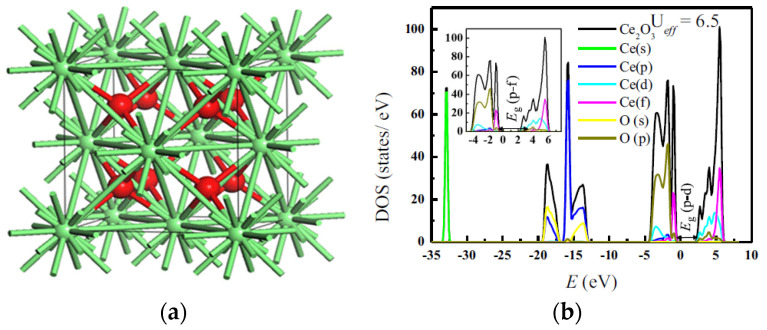
(**a**) Cubic fluorite structure of the lanthanide dioxide. (**b**) The theoretically predicted total and partial density of states for Ce_2_O_3_ sesquioxides [14].

Promising agreement was obtained between the theoretical and empirical studies for the investigated properties. The reported findings indicated that the inclusion of Hubbard parameter U in the methodology resulted in a noticeable correction to the structural properties. The theoretically calculated bulk moduli B, its pressure derivative B′, and the phase transition pressures are compared with some experimental results and depicted in Table 1. The influence of including Hubbard parameter DFT+U on assessing the structural, electronic, and thermomechanical properties of cubic (C-type) lanthanide sesquioxides (Ln_2_O_3_) has been reported [14]. Figure 2b portrays the total and partial density of states with conduction and valence bands clearly shown. Besides, the plot displays that the experimental value of the band gap is obtained at U_eff_ of 6.5 eV. Regarding the negative values in Table 1, they indicate that the C-type phase is the preferred (most stable) phase.

**Table 1:** Bulk modulus B, its pressure derivative B′, and the phase transition pressure for the complete series of Ln_2_O_3_. Reproduced from [12].

Compound	Functional	*A*-Phase		*C*-Phase		P_C→A_ (GPa)
		B (GPa)	B′	B (GPa)	B′	
**La_2_O_3_**	LDA GGA + *U* Exp. [15]	155.2 142.8 113	4.34 4.39 6.0	133.9 124.4	4.15 4.18	0.0
**Ce_2_O_3_**	LDA GGA + *U*	166.8 142.0	4.45 4.29	148.5 135.5	5.62 4.00	−2.6
**Pr_2_O_3_**	LDA GGA + *U*	170.6 152.3	4.38 4.00	148.2 157.9	4.46 4.00	−3.9
**Nd_2_O_3_**	LDA GGA + *U*	173.5 155.1	4.43 3.62	150.5 122.0	4.38 5.45	−3.7
**Pm_2_O_3_**	LDA GGA + *U*	176.2 156.1	4.50 4.01	153.8 129.0	4.50 4.00	−2.7
**Sm_2_O_3_**	LDA GGA + *U* Exp. [15]	177.4 147.0 130	4.42 4.49 6.9	153.4 138.3 116	4.22 4.29 4.0	−1.0
**Eu_2_O_3_**	LDA GGA + *U* Exp. [15]	177.7 134.3 134.0	4.39 4.00 4.1	156.1 143.1 115	4.33 4.17 5.9	0.5
**Gd_2_O_3_**	LDA GGA + *U* Exp. [15]	178.1 160.7 145.0	4.32 4.39 4.2	158.3 144.7 125.0	4.42 4.24 4.7	−0.7
**Tb_2_O_3_**	LDA GGA + *U*	179.5 159.9	4.22 4.53	158.6 139.0	4.31 4.67	−0.3
**Dy_2_O_3_**	LDA GGA + *U*	180.9 160.4	4.24 4.64	159.9 148.9	4.37 5.14	1.5
**Ho_2_O_3_**	LDA GGA + *U* Exp. [16]	180.9 179.1 204	4.63 3.71 3.8	161.6 152.0	4.50 4.48	3.4
**Er_2_O_3_**	LDA GGA + *U*	180.4 173.6	4.64 4.51	161.2 157.2	4.46 3.98	5.7
**Tm_2_O_3_**	LDA GGA + *U*	178.5 168.4	4.56 4.65	161.6 157.7	4.40 4.36	7.0
**Yb_2_O_3_**	LDA GGA + *U*	177.8 178.7	4.61 4.33	161.6 160.9	4.52 4.27	7.5
**Lu_2_O_3_**	LDA GGA + *U*	198.8 179.9	4.33 4.29	179.4 163.0	4.30 4.29	7.7

The oxidation of cerium metal is known to result in the formation of many different phases of cerium oxide ranging from CeO_2_ (IV) to Ce_2_O_3_ (III). The oxidation processes of these two extreme oxidations are exothermic by −1796 and −1089 kJ mol^−1^, respectively, at a temperature of 298 K [17] Between these two phases, partially oxidized phases (CeO_2−y_) prevail depending on the temperature and oxygen pressure. Ceria exhibits a fluorite structure crystallized as a centered cubic face (fcc) with the space group of Fm-3m (a = 0.541134 nm, JCPDS 34–394), comprising a cubic close-packed combination of metal atoms with tetrahedral holes filled by oxygen atoms. Reduced CeO_2−y_ forms by releasing oxygen atoms from the cluster, leaving oxygen vacancies behind. The Kröger–Vink notation governs the process of creation of vacancies as follows [17]:

(1) $$2{\mathrm{Ce}}_{\mathrm{Ce}}+O_{O}\;\to V_{O^{¨}}+2{\mathrm{Ce}}_{\mathrm{Ce}}^{\prime }+\frac{1}{2}O_{2}$$

where Ce and O denote cerium and oxygen atoms, respectively, and V_Ö_ signifies oxygen vacancy. On the basis of the notation above, the exact nature of the resultant phase relies on the amount of oxygen released from CeO_2_. X-ray diffraction (XRD) was employed to determine the structural parameters of reduced CeO_2−y_ oxides, but this technique exhibits some limitations due to the low scattering power of oxygen. As Table 2 portrays, at temperatures over 685 °C and low oxygen pressure, CeO_2_ exhibits several forms of nonstoichiometric oxidation states (CeO_2−y_).

With y ranging from 0 to 0.286, a disordered structure of a fluorite-related system termed the α phase develops [18]. All phases in this oxidation range adopt a fluorite-type structure but with an ordered arrangement. Formed phases include [19,20,21,22] Ce_6_O_11_ (the β phase, monoclinic) [22], Ce_11_O_20_ (the δ phase, triclinic) [23], and Ce_7_O_12_ (rhombohedral) [23]. When y increases to exceed 0.286, a new phase termed the σ phase emerges. The σ phase exists as a body-centered cubic (bcc) structure. The C-type sesquioxide Ce_2_O_3_ formed in the bixbyite structure (space group Ia-3) is the compositional final member of the σ phase which is related to the fcc structure of CeO_2_. The lattice parameters of the C-type Ce_2_O_3_ are nearly twice those of CeO_2_. This is attributed to the two cation groups being nearly identical, with oxygen anions residing in all tetrahedral sites in the fcc structure, whereas only three-quarters exist in the bcc structure in an ideally ordered array. Due to the high reactivity of the cubic sesquioxide structure (C-type Ce_2_O_3_) [24] with atmospheric oxygen, the final crystal structure, called the θ phase, is formed. This phase is well known as the A-type Ce_2_O_3_ which is crystallized in a hexagonal structure form, belonging to the P32/m space group (a = 0.389 nm, c = 0.607 nm; JCPDS 23-1048) [25].

## 3. Recent Computational Modeling Based Literature

It is worth mentioning that from an atomistic point of view, numerous density functional theory (DFT)-based studies with various functionals such as the HSE06 (Heyd–Scuseria–Ernzerhof hybrid functional) and the DFT+U approach (U corresponds to Hubbard parameter describing the on-site Coulomb interactions) have been reported. In reference to corresponding experimental studies, DFT investigations accurately predict chemical bonding, vacancy-defect formation, band structures, surface character, thermomechanical properties, and doping effect. The defective nature of CeO_2_ (111) has been demonstrated under the framework of DFT with several functionals. It is generally suggested that two excess electrons are localized on the next-nearest neighbors, not on the nearest neighbors [26]. A combination study employing scanning tunneling microscopy (STM) and DFT calculations has been carried out to identify the synergy between an oxygen vacancy and the associated Ce^3+^ ion pair in a defective CeO_2_ (111) plane. The two Ce^3+^ ions can occupy different cationic shells around the vacancy. Both reported results reveal that at least one excess electron localizes in a Ce ion that is not next to the O vacancy [27].

Moving toward the thermodynamic aspect, Fronzi et al. [28] employed an ab initio atomistic thermodynamics approach to assess relative thermodynamic stability and Wulff constructions of the three low-index surfaces of CeO_2_, namely (100), (110), and (111). Among the studied surfaces, the stoichiometric (111) surface under “oxygen-rich” conditions is predicted to be the most stable surface. Under reducing conditions, the stoichiometric (111) face with subsurface oxygen vacancies becomes the most thermodynamically stable facet. However, near the O-lean region, the (111) Ce-terminated surface becomes the most stable surface. A combination of DFT results and Monte Carlo simulations was achieved to investigate the dopant dispersion and its impact on the oxygen ion conductivity of ceria alloyed with rare earth oxides [29]. The consensus in the literature suggests that an accurate description of the electronic system of CeO_2_ requires the inclusion of the U term in the DFT calculations. Pure DFT methods incorrectly describe ceria as a conducting material. DFT-based investigation revealed that in the near-surface region of CeO_2_ (111), at low temperatures and vacancy concentrations, subsurface oxygen vacancies showed more stability than surface ones [30].

## 4. The Role of Dopants Introducing on CeO_2_ Properties

**Figure 3:**
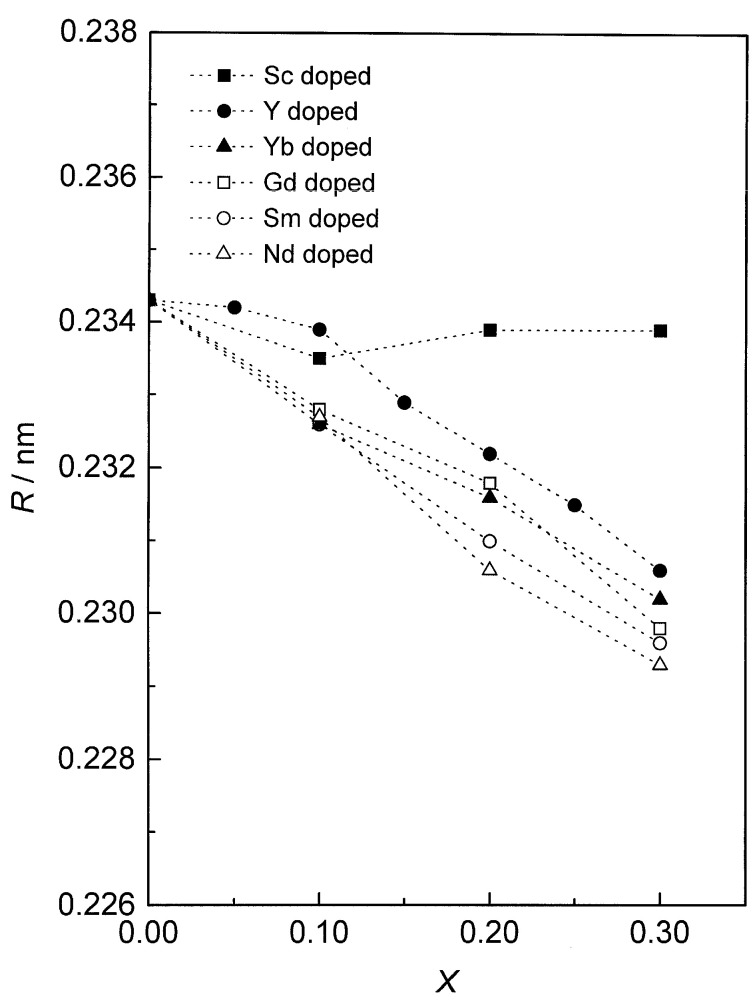
Trends of the Ce–O interatomic distances in ceria doped with Sc, Y, Yb, Gd, Sm, and Nd as a function of dopant concentration x [31].

It has been reported that the addition of various dopants into the crystallite structure of ceria would enrich thermal and chemical stability and lead to strong ultraviolet (UV) absorption of the crystal. Thus, when introducing an element with an oxidation number lower than that of cerium, oxygen is removed, forming oxygen vacant sites in the ceria structure. This suggests that the defect chemistry of ceria lattice and structural changes can be produced by doping. Defect structures and changes in the lattice as a function of dopant concentration in doped CeO_2_ have been probed using extended X-ray absorption fine structure (XAFS) and X-ray absorption near-edge structure (XANES) spectroscopies [31]. XAFS data for doped ceria for various dopants at several concentrations (Ce_1−x_ Ln_x_O_2−x/2_ (Ln refers to Sc, Y, Nd, Sm, Gd, Yb, x = 0–0.30)) show that interatomic distance decreases with increasing Ln concentration, suggesting that the oxygen ions are relaxed toward oxygen vacancies around Ce, Y, and Gd. The decrease in the Ln–O interatomic distances can be explained by the formation of the defect correlated with two Ln^3+^ ions and one oxygen vacancy and/or four Ln^3+^ ions and two oxygen vacancies. Figure 3 depicts the reduction in interatomic distances with increasing dopant fraction which is associated with increasing disorder. This signifies that the oxygen ions are accumulated near oxygen vacant sites nearby Ce, Y, and Gd. The reduction trend of the Sc–O interatomic distances is almost constant with increasing concentration due to the existence of two phases of fluorite (CeO_1__−x_ Sc _x_ O_2__−x/2_) and Sc_2_O_3_ structures. In the same manner, when introducing dopants larger than ~102 pm, such as Gd^3+^ and Sm^3+^, the average lattice parameter in the fluorite phase as obtained using X-ray diffraction technique increases with increasing doping concentration [32].

Previous works on CeO_2_ and CeO_2_-based materials concluded that dopant introduction induces lattice distortion that in turn leads to positional disorder and atomic displacement of constituent atoms. Furthermore, introducing elements with less than 4^+^ valence electrons, such as Ca^2+^, Nd^3+^, and Pb^2+^, would promote structural defects (i.e., oxygen vacancies) in the ceria lattice, which in turn impact the redox activity of these materials [33]. Another study reported that introducing trivalent lanthanide ions in ceria results in distorting the lattice constant and thereafter forming oxygen vacancies by replacing the 4^+^ site, which is crucial in catalytic reactions [34]. The luminescence property of CeO_2_ has significantly improved by incorporating lanthanide ions in the lattice structure. This has been noticed via variant types of obtained luminescence peaks, linked with oxygen vacancies and dopant types causing symmetry distortion [35]. Practically, this distortion in the structures can be determined by Raman spectroscopy via testing the factors influencing the line shape, width, and position of the Raman peaks of doped and pure ceria. Furthermore, literature has nominated Y^3+^-doped CeO_2_ as an efficient system in converting wavelengths of photons near the UV to IR range [36].

Moreover, RE elements, such as La, Ce, and Y or their oxides, would enrich the high-temperature oxidation resistance of alumina and chromia alloys via an enhancement of their reactive-element effect (REE) [37,38]_._ Thanneeru et al. [39] coated AISI 304 stainless steel (SS) with nanocrystalline ceria and La^3+^-doped nanocrystalline ceria particles with the aim of studying their high-temperature oxidation resistance at 1243 K in dry air for 24 h. Results were then compared to those of similar coatings in the absence of microceria coatings. The nanocrystalline ceria coatings were observed to enhance the oxidation resistance character by 90% compared to those cases of uncoated and microceria-coated steels. Likewise, Fernandes and Ramanathan [40] reported the effect of surface coatings of Ce, La, Pr, and Y oxide gels on the oxidation behavior of a Fe-20Cr alloy at 1000 °C. Alloying small quantities of rare earth elements to chromia or alumina enhances their high-temperature oxidation resistance. Usually, rare earth elements are added or doped with oxides to form a protective layer for chromia and alumina alloys. In addition, it is important to mention that the morphology of RE oxide coatings varies with the nature of the RE. For instance, the oxidation rate of RE oxide coated Fe-20Cr was considerably less than that of the uncoated alloy. The influence of Mn and Fe doping into the CeO_2_ (111) surface on the simultaneous removal of mercury (Hg) and H_2_S was examined under the framework of DFT. In this study, the adsorptions of Hg-containing species on perfect CeO_2_ (111), Mn/CeO_2_ (111), and Fe/CeO_2_ (111) surfaces were investigated. The results showed that Mn and Fe dopants expedited Hg adsorption [41]. Besides, in this work, the redox activity of a low praseodymium (Pr)-doped CeO_2_ (111) surface was examined via DFT. Findings reveal that Pr doping stimulates oxygen donation by dropping the required energy to produce surface anionic vacancies [42].

## 5. Solid Solutions and the Influence of Reduction Energies of Ceria

**Figure 4:**
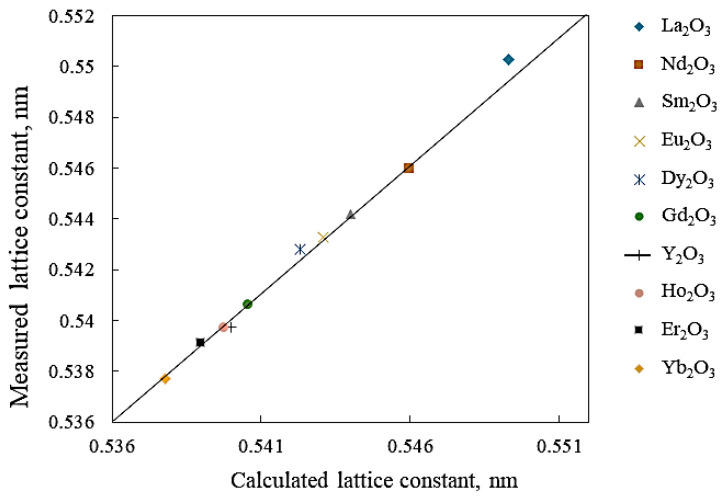
Experimental and calculated lattice parameters of fluorite-structure CeO_2_ solid solution containing variant rare earth sesquioxides [43].

It is well known that the fluorite structure of CeO_2_ has the capability of forming solid solution systems with a wide array of oxides. The lattice dimensions of the solid solution typically obey Vegard’s rule, i.e., a linear relation between lattice constant and solute concentration. It must be emphasized that the term “dopant” should be utilized for cases involving the introduction of a foreign cation in the ceria lattice, as opposed to situations in which two oxides are mixed [17]. Kim [43] reported an empirical equation clarifying the relationship between the lattice parameters of the solid solution, along with the ionic radius and the cation charge of the dopant introduced into the CeO_2_ and fluorite-like oxide structures. The relation is expressed as follows:

(2) $$a=0.5413+\sum_{k}(0.0220∆r_{k}+0.00015∆z_{k})m_{k}$$

where *a* (in nm) signifies the unit cell constant of the solid solution containing CeO_2_, $∆r_{k}=r_{k}-r_{Ce\;\left(IV\right)}$ in nm corresponds to the variance between the *k*th dopant and Ce (IV) ionic radii, $∆z=z_{k\;}-z_{Ce\;\left(IV\right)}$ in nm denotes the charge variance of the *k*th dopant and Ce (IV), and m_k_ signifies the molar focus of the *k*th dopant. Kim [44] indicated that the solubility of either oxide material into the fluorite crystallographic structure of CeO_2_ relies on the elastic energy per ion present into the unit cell due to the variance in ionic radius. Therefore, a greater magnitude of ∆*r_k_* drives a higher elastic energy and a lower solubility limit. The most soluble cations possess a radius that corresponds to the matching radius, *r_m_*, the one that results in Vegard’s slope being equal to 0. According to Kim’s equation, *r_m_* must have a value of 0.097 nm for tetravalent dopant cations, 0.1038 nm for trivalent dopants, and 0.1106 nm for divalent dopants. Similar amounts were reported in previous works [44,45]. Below, Figure 4 displays the measured and computed lattice parameters of fluorite-structure CeO_2_ solid solutions formed with different rare earth oxides. CeO_2_-ZrO_2_ mixed oxides have received significant attention in literature because of their wide deployment in the so-called three-way catalysts (TWCs). As such, the structural properties of CeO_2_-based solid solutions have been thoroughly investigated. The difference between the ionic radius of Zr^4+^ (0.084 nm for a 8-fold coordination) and [46] that of Ce^4+^ (0.097 nm) is only 15%. In addition, metal oxide solid solutions, e.g., CeO_2_-ZrO_2_, have received significant attention in literature because of their wide deployment in the so-called three-way catalysts (TWCs).

**Figure 5:**
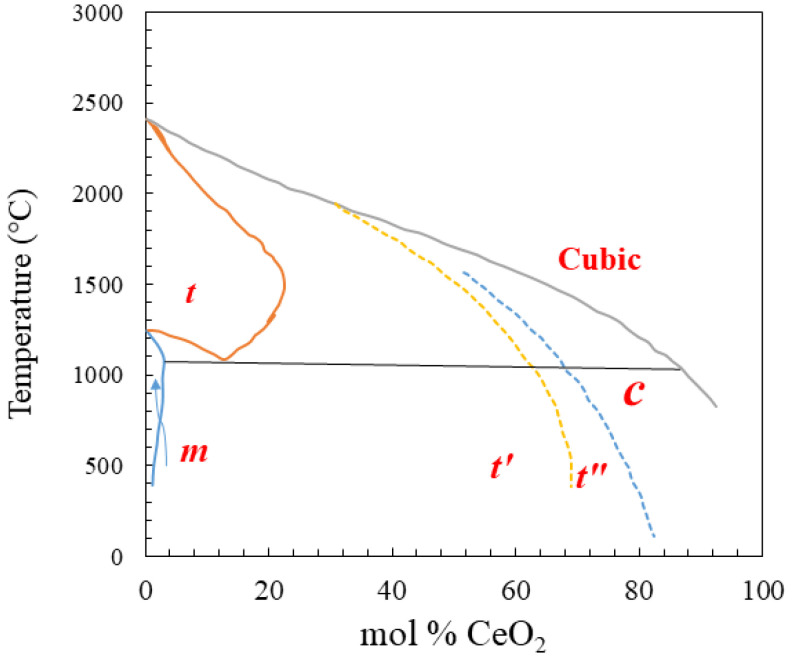
Phase diagram study of CeO_2_-ZrO_2_ system with different CeO_2_ concentrations as a function of temperature. t, t′, and t″ demonstrate the three forms of the tetragonal phases, m refers to the cubic phase, and m denotes the monoclinic phase [48].

Yashima and his colleagues [47] conducted studies on the CeO_2_-ZrO_2_ solid solution annealed in a Na_2_B_2_O_7_-NaF atmosphere. They studied the properties of the system below 1000 °C using XRD analysis, displaying the entire equilibrium phase diagram of the CeO_2_-ZrO_2_ solid solution. Analysis of their phase diagram reveals three crystalline structures depending on the temperature [47], namely cubic phase with high CeO_2_ percentage and tetragonal or monoclinic phases with high ZrO_2_ percentage. The balanced compositions of the tetragonal, monoclinic, and cubic phases occur at x = 0.112, 0.009, and 0.84 in Ce_x_Zr_1−x_O, respectively, at a temperature of 1055 °C [47], as depicted in Figure 5.

Detailed interpretation of the phase diagram is that a monoclinic crystalline phase belonging to the P2_1_/c space group is obtained for ZrO_2_ and Ce_x_Zr_1−x_O_2_ system at x values lower than 0.12. With the increase in CeO_2_ concentration in the system, the a_m_ (m refers monoclinic) value approaches that of b_m_ and the angle β_m_ lessens, indicating a distortion of the monoclinic phase, and all approach those of the tetragonal structure [49,50]. It must be noted that the phase boundary of x = 0.12 is substantially affected by some parameters such as the preparation of the sample and the grain size. These two parameters in turn affect the nucleation, growth, and kinetics of the transformation [50]. The three tetragonal phases denoted as t, t′, and t″ [51,52] are crystallized when the oxygen content becomes higher than 0.12, and at a monoclinic phase, the O content would be so low as 0.12. The t phase is stable at elevated temperature and for lower CeO_2_ concentrations. As the CeO_2_ content increases, the other two metastable phases of t′ and t″ are formed. For the t and t′ forms, the c/a ratio is slightly higher than 1, whereas the ratio of the t″ phase belonging to the P4_2_/nmc space group equals 1.

According to the vacancy formation energy in a CeO_2_ cluster calculated via DFT, for systems with dopant amounts of 3 mol%, tetravalent dopants such as Ti, Zr, and Hf (IVb in the periodic table) can be inserted into the bulk of CeO_2_. On the contrary, other elements such as C, Si, Ge, Sn, and Pb (IVb in the periodic table) are segregated on the surface [53]. It is found that the vacancy creation energy of 4.035 eV per vacancy calculated by LDA functional and 3.097 eV per vacancy obtained by PBE functional are reduced with increasing dopant size, reaching the best size that matches the Ce^4+^ ions.

CeO_2_-HfO_2_ samples have been the subject of extensive structural investigations. Findings obtained from these studies revealed similar structures to those observed for the CeO_2_-ZrO_2_ system. On the basis of XRD studies [54] for samples annealed at 1400 °C for 48 h and cooled down slowly, stable Ce_x_Hf_1-x_O_2_ solid solutions with x > 0.85 (CeO_2_-rich materials) are crystalized with cubic fluorite structure. Meanwhile, when x < 0.15 (HfO_2_-rich materials), solid solutions are formed with monoclinic structure. The samples characterized by a combination of XRD and Raman analysis adopted the metastable tetragonal phases (t′ as well as t″) [55].

## 6. Influence of the Reduction Energies of CeO_2_

**Figure 6:**
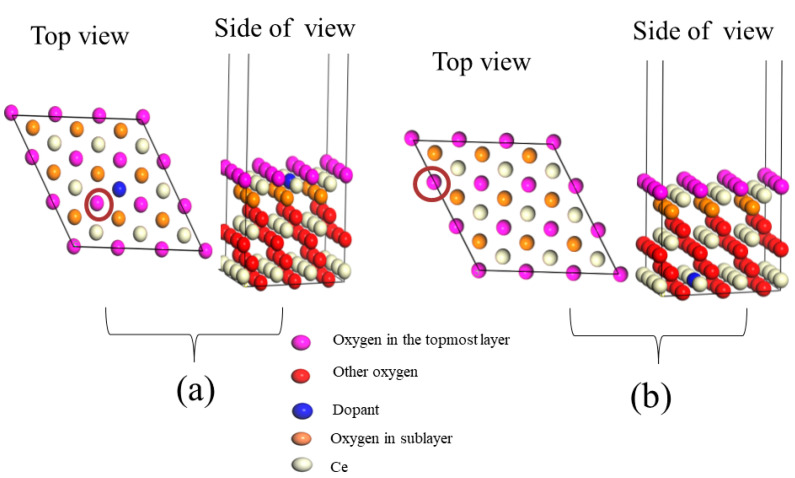
Side and top views of CeO_2_ (111) slab, doped with a dopant. (**a**) Removal of neighboring oxygen in the doped slab. (**b**) Removal of furthest oxygen in the doped slab [57].

It is important to investigate the redox properties of a catalyst by calculating its reduction/oxidation energy. This in turn helps to predict the capability of that material in performing catalytic oxidation reactions. This investigation is carried out by calculating the vacancy formation energy E_vac_ needed to remove oxygen from the system for oxidation and hence create a vacancy. An improvement by lowering the reduction energies of oxides is achieved via replacing the cations of the catalyst with others [56,57,58]. DFT investigations undertaken by Hu and Metiu [57] studied the influence of the addition of various cations to CeO_2_ catalyst on its reduction energy. In this study, the authors surveyed the effect of adding some dopants such as Pt, Ru, Zr, Ta, Mo, and W dopants in CeO_2_ (111) on the neighboring oxygen or distant ones. For the neighboring oxygen atoms, the calculated energies of oxygen vacancy creation caused by the added cations (added dopants) were found to be almost identical. By contrast, an effect of these dopants on the reduction energy of the distant oxygen has not been recorded. Figure 6 displays the removal of oxygen atoms from different positions in the CeO_2_ (111) slab as a result of introducing dopants.

In another instance, first-principle calculations reported by Yang et al. [59] surveyed the impact of introducing Zr into a CeO_2_ system on the redox properties of CeO_2_. They reported that the reduction energy needed to remove a neighboring oxygen atom was reduced by 0.6 eV in reference to undoped CeO_2_. In a separate work, Yang and his collaborators [60] evaluated the vacancy formation energy of Pd-alloyed CeO_2_. They concluded that introducing Pd atoms into the ceria system lowered the vacancy formation energy from 3.0 to 0.6 eV. To acquire an accurate understanding of the role of trivalent and tetravalent cations such as Zr^4+^, La^3+^, and Eu^3+^ incorporated into the ceria lattice, Vinodkumar et al. [61] used DFT calculations to reveal that alloyed ceria materials exhibit a better efficiency than unalloyed ceria for soot oxidation and this is attributed to an enhancement in the oxygen defects, specific surface area, and redox properties. Under the exposure of air under tight contact conditions, trivalent-alloyed CeO_2_ was proven to be more efficient than tetravalent-alloyed ceria for soot combustion. Finally, Eu^3+^-doped CeO_2_ has been demonstrated to be catalytically more active than La^3+^–doped CeO_2_, and this was ascribed to higher surface area and an increase in oxygen vacancies. Kim et al. [62] assessed the thermodynamic characteristics of Ce_1−x_ Zr_x_ O_2−y_ solid solutions.

## 7. Ceria Surface Reactions with Inorganic Molecules

### 7.1. Interaction of H_2,_ O_2,_ and H_2_O with Ceria

Hydrogen molecules (H_2_) have been reported in many studies as a reducing agent for CeO_2_ powders at high temperature and at atmospheric pressure [63,64]. Results obtained by experimental work revealed that H_2_ cannot adsorb or react with CeO_2_ surfaces under ultrahigh vacuum (UHV) conditions [65,66,67,68]. Furthermore, DFT-based investigations provided potential energy surfaces for the dissociative uptake of hydrogen molecules over CeO_2_ (111) and CeO_2_ (110) surfaces via exothermic reactions [69,70]. It has been proven that at low exposure of nonreduced CeO_2_ (111) to D atoms at 115 K, surface OD is formed. As a consequence of this reaction, Ce^4+^ states are reduced to Ce^3+^ states. A comprehensive review on the basis of XPS analysis revealed that at greater coverage (>50 L), water is detected in a high-resolution XPS examination of O 1 s photoelectron, suggesting that D(g) (g meaning gas) adsorbed on the surface reacts with OD to produce chemisorbed D_2_O. The surface OD group reacts with D on the surface to form D_2_O(g) between 200 and 600 K. It is demonstrated that the chemisorbed D_2_O(g) molecules are desorbed at temperature close 200 K, whereas D_2_ desorbs between 400 and 500 K [65]. Exposing reduced CeO_2−y_ (111) to D(g) formed OD on the surface, but the trend of producing D_2_O at higher exposure lessens with further reduction of the surface. It has been observed that the stability of the OD formed on reduced CeO_2−y_ (111) is greater than that of pristine CeO_2_ (111). For the different CeO_2−y_ (111) configurations, water and D_2_ were found to desorb at 570 K. From a theoretical standpoint, DFT studies on the adsorption of H_2_(g) on CeO_2_ surfaces have reported that dissociative uptake of H_2_(g) O sites in CeO_2_ (111) and CeO_2_ (110) surfaces result in the partial reduction of neighboring Ce cations [71]. Another theoretical approach of ultra-accelerated quantum chemical molecular dynamic simulations demonstrated a mechanism for the release of water molecules following adsorption of H_2_ molecules [72]. The high oxygen storage of ceria renders it a favorable material for wide deployment in the TWC technology in vehicles.

**Figure 7:**
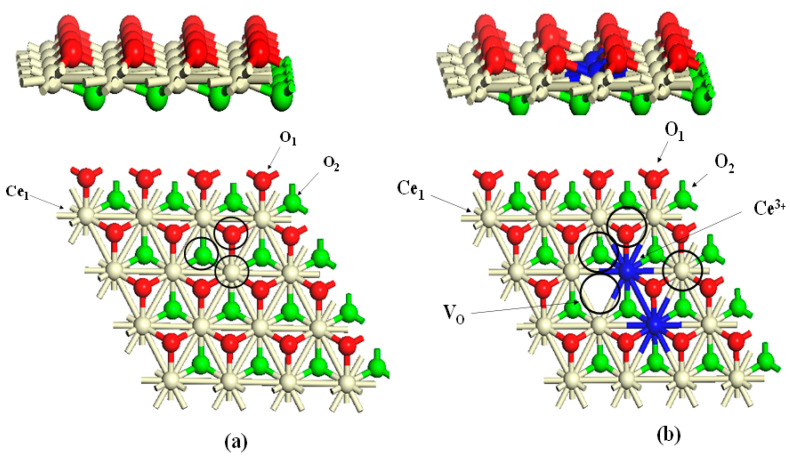
Active sites on CeO_2_ (111) surface: (**a**) side and top views of perfect CeO_2_ (111) surface; (**b**) side and top views of reduced CeO_2_ (111) surface.

As stated earlier, CeO_2_ acts as an oxidizing agent in fuel-rich/oxygen-deficient periods and as a reducing agent in the oxygen-rich periods. Consequently, it is crucial to understand the physisorption and chemisorption reactions of O_2_ occurring on CeO_2_ surfaces. It has been reported that the oxidation of Ce metal occurs at a temperature of 300 K, leading to a form of Ce_2_O_3_ covered by a layer of CeO_2_ [73]. When the polycrystalline structure was heated to 600 K, XPS examinations of the Ce 3d and Ce 4d photoelectrons peak showed complete reduction to Ce_2_O_3_. In contrast, reducing a CeO_2_ (100) surface by Ar^+^ ion sputtering at 300 K displayed partial reoxidation as it is annealed to 600 K in vacuum [66]. These two findings indicate that O is redistributed between the surface and the bulk below 600 K. Along the same line of inquiry, DFT calculations were used to determine reaction routes for the interaction of O_2_ with stoichiometric and reduced ceria surfaces. Dissociative adsorption of oxygen molecules over the CeO_2_ (111) surface is predicted to be endothermic with values residing on the range of 0.91–0.98 eV [74]. In another DFT+U study, reactions of O_2_ with the partially reduced CeO_2−y_ (111) surface promote superoxo species bonded weakly to its surface O-vacancy (−0.30 to −0.38 eV) and peroxo species that are more strongly bonded (−2.80 to −3.25 eV) [75]. In another related study on the partially deficient CeO_2−y_ (110) and CeO_2−y_ (100) surfaces, binding of the peroxo species was found to involve an adsorption energy of −2.0 eV [76]. Experimental work on the adsorption of H_2_O on CeO_2_ (111) and CeO_2_ (100) demonstrated that H_2_O can be physisorbed, chemisorbed, or both depending on the applied temperature [77,78,79]. This is consistent with analogous computational results for CeO_2_ (111). For instance, Fronzi et al. [80] and Marrocchelli and Yildiz [81] concluded in separate studies that H_2_O preferentially adopts several physisorbed states on CeO_2_ (111), whereas Watkins et al. [70] illustrated that water fragmentation into H and OH is a feasible process. Molinari et al. [82] studied the adsorption of water on the most stable surfaces, CeO_2_ (111), (100), and (110), employing DFT-GGA-U calculations. They reported that H_2_O molecules readily dissociate on the studied surface and that the most stable surface towards the chemisorbed H_2_O is the CeO_2_ (111) surface. They further concluded that the molecular adsorption of water becomes more preferable when the water coverage increases. Fernandez-Torre et al. used different DFT calculations to estimate energy barriers for the steps governing water adsorption where they reported very similar energy barriers for the different steps [83]. As stated earlier, ceria fluctuates between two extreme oxidation states of 4^+^ and 3^+^, and the reduction occurs via the releasing of oxygen atoms from CeO_2_. As such, the creation of oxygen vacancies as a result of CeO_2−y_ (111) surface undergoing a reduction reaction leads to the production of Ce cations with two coordination vacancies and three-fold hollow adsorption sites [84]. Below, Figure 7 illustrates the active sites on the CeO_2_ (111) surface.

A number of computational-based studies have reported similar conclusions that vacancies created on CeO_2−y_ (111) surface enhance and stabilize the decomposition of H_2_O. A survey of the literature suggests that the reaction between -OH on CeO_1.7_ (111) leads to the release of H_2_. It must be noted that H_2_ desorption following adsorption of water on CeO_1.7_ (111) is contradictory to the desorption from reduced CeO_1.7_ (100) in which the primary desorption route remains recombination to form water with only a trivial amount of H_2_ formation. The energy of vacancy creation on CeO_2_ (111) is found to be greater than that on CeO_2_ (100). Hence, it is expected that the driving force to fill the vacancy is greatest on CeO_2−y_ (111) surfaces.

**Figure 8:**
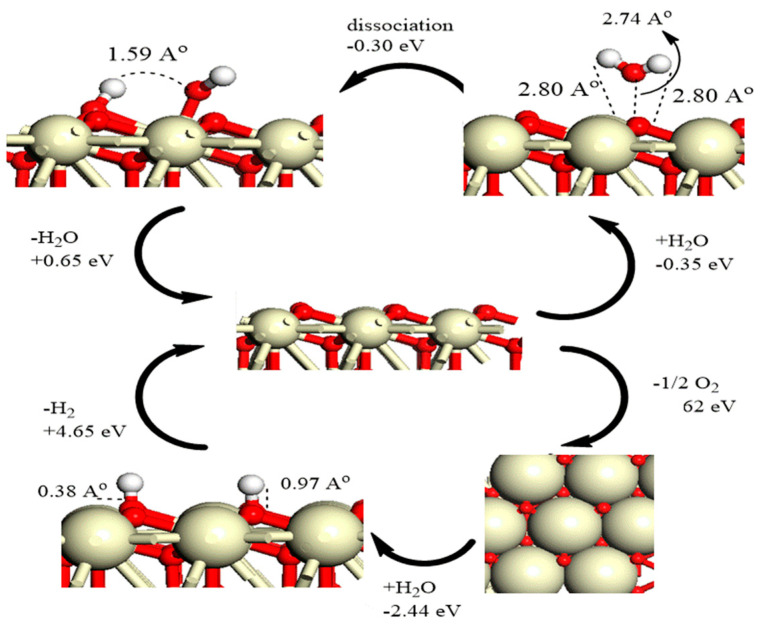
H_2_O and H_2_ reactions on the defective and perfect ceria (111) surfaces [70].

The decomposition pathways of H_2_O and H_2_ on both the pristine and defective ceria (111) surfaces have been assessed by means of DFT+U method [70]. The H_2_O physisorption reaction is an exothermic process on both configurations (see Figure 8); however, the physisorption of water is more exothermic on the vacancy site. The dissociation of water on the two studied surfaces results in the formation of two hydroxyl groups, one in the initial water physisorbed site and the other on a surface oxygen ion. Furthermore, Watkins and coworkers [70] reported a potent exothermic chemisorption reaction for H_2_ on the perfect ceria (111) surface, which is ascribed to the low-lying 4f states, and thereafter great electron affinity of ceria. Another study concluded that the decomposition of water is preferred on the perfect surface of ceria (111) due to the formation of a strong hydrogen bond between the OH^−^ and H^+^ moieties created upon decomposition. It also has been demonstrated that H_2_O can strongly bind the CeO_2_ (111) surface over O vacant sites [81].

### 7.2. Sulfur Dioxide (SO_2_)

It is understood that trace concentration of sulfur-based molecules in fossil fuels results in the production of sulfur oxides (SO_x_) in the exhaust. Adsorption of such molecules on ceria has a negative effect on its oxygen storage capacity properties in automotive catalytic convertors. However, the high affinity for SO_x_ can, in principle, be exploited by trapping sulfur in the effluent gases. SO_2_ adsorption on cerium oxide thin films has been investigated by two studies, and two different conclusions were reached. Through surface measurements on a SO_2_-CeO_2_ (111)/Ru (0001) system, Overbury et al. [85] concluded that SO_2_ is adsorbed as sulfite ion $SO_{3}^{2-}$ on the stoichiometric surface at temperatures from 100 to 600 K. According to S2p high-resolution XPS spectra, the SO_2_ adsorbs via a Lewis acid–base interaction at the basic $O^{2-}$ surface sites. SO_2_ molecularly desorbs with main desorption peaks close to 200 and 400 K. There was no evidence to suggest that the oxidation process yield $SO_{4}^{2-}$ or the reduction process yields $SO^{2-}$ or $S^{2-}$, which is in agreement with a previous study [86]. They utilized vibrational spectroscopy to study CeO_2_ powders, observing that only $SO_{3}^{2-}$ forms after SO_2_ is exposed to room temperature [87]. Sulfate formation occurs following exposure at 673 K and is encouraged by simultaneous exposure to O_2_. Contradictory to these studies, Rodriguez et al. [88] observed that SO_2_ was adsorbed almost exclusively as $SO_{4}^{2-}$ on stoichiometric, polycrystalline CeO_2_/Pt (111). To elucidate the contradiction, a number of additional experiments were performed in which different parameters were assessed. The adsorption of SO_2_ on polycrystalline CeO_2_ films deposited on Al_2_O_3_ utilizing an SO_2_ pressure of 2.5 mbar was studied by Smirnov et al. [89]. In their study, they demonstrated $SO_{3}^{2-}$ formation at temperature below 473 K and $SO_{4}^{2-}$ formation above 573 K. As O_2_ gas with an identical pressure was introduced alongside SO_2_ exposure, $SO_{3}^{2-}$ formation was suppressed at the low temperatures, but $SO_{4}^{2-}$ was still apparent at the higher temperatures. Interestingly, the sulfate concentrations were enhanced by the introduction of O_2_ at higher temperatures. Likewise, Ferrizz and his coworkers used polycrystalline ceria films deposited on Ta foil by spray pyrolysis to carry out TPD measurement. In their XPS examinations, they utilized a different substrate, Mo (100), to deposit CeO_2_ in an O_2_ atmosphere synthesized by Ce vapor deposition. After SO_2_ introduction at 298 K, a major SO_2_ desorption peak was evident at 473 K [90], which agreed well with analogous literature findings [85]. However, another SO_2_ desorption peak was noted residing in the range of 800–1000 K, which was not observed in their results [85]. As SO_2_ exposure temperature increases to 573 K, the intensity of peaks located at higher temperatures increases, unlike those in the lower temperature range that start to decay. The S2p photoelectron line obtained by XPS suggested that the SO_2_ adsorbed mainly as sulfite at 298 K but some of this transformed to sulfate when the sample was annealed. Increasing the exposure temperature to 923 K resulted in more transformation of the sulfate into sulfide. Analogous findings were reported with the pure SO_2_ exposure and as a mixture with O_2_.

The adsorption of SO_2_ has been carried out over CeO_2_ (111)/Cu (111) [91]. The XPS analysis suggested that sulfite is the main surface species at 300 K. When the sample was annealed, some of the sulfur was reduced to $S^{0}$ and $S^{2-}$. There was no sulfate observed at any temperature. The physisorption and chemisorption reactions of sulfur dioxide (SO_2_) were investigated on single-crystal metals such as Cu, Au, and Pt and on pure CeO_2_ (111), as well as on the metal-supported forms of CeO_2_ (111) such as Cu-supported, Au-supported, and Pt-supported CeO_2_. In these previous investigations, SO_2_ adsorption and dissociation on CeO_2_ (111) doped with Cu, Au, and Pt results in different products. For instance, it was found that SO_2_ interacts with the surface oxygen on CeO_2_ (111) to produce the sulfite ion $SO_{3}^{2-}$ or sulfate ion $SO_{4}^{2-}$. At higher temperatures, $SO_{3}^{2-}$ and $SO_{4}^{2-}$ were found to be desorbed without dissociating as S^0^ or S^2−^ on the catalyst surface. The reaction of SO_2_ occurs spontaneously in the case of metal-based CeO_2_ (111). At over 250 K, SO_2_ has been observed as a molecule on Au/CeO_2_ (111) without decomposing. On Cu/CeO_2_ (111), the dissociation to S^0^ is improved by contrast to Cu single crystals. The activity peaks at Cu coverage ranging from 0.5 to 1.0 ML, and beyond this the activity decays back. However, the overall reaction of SO_2_ on Pt/CeO_2_ (111) catalyst is rather complicated. At a temperature of 150 K, on the CeO_2_ (111) surface, SO_2_ adsorbs as sulfite standing upright on the Pt nanoparticles depending on the XPS high-resolution S2p photoelectron line. This is in line with those cases of metal-free CeO_2_ (111) and Pt (111), except for the absence of SO_2_ lying flat on the Pt nanoparticles.

### 7.3. Nitrogen Oxide (NOx)

Given the importance of ceria’s catalytic performance for treating NO_x_ in automobile exhaust, the adsorption of nitrogen oxide in its forms as N_2_O, NO, and NO_2_ on ceria surfaces has been investigated [92]. Ferrizz et al. [93] indicated the nonadsorption of NO at 300 K on the fully oxidized CeO_2_ (111) single crystal or on CeO_2_ deposited on α-Al_2_O_3_ (0001). Overbury et al. [94] further reported that NO does not interact with CeO_2_ (100) at 160 or 300 K; however, NO was observed to interact rather weakly at 90 K on stoichiometric CeO_2_ (111)/Ru (0001) [94]. Literature reported that NO is physisorbed below 200 K with small amounts of N_2_O and N_2_ desorbing in the same temperature window. The wide temperature range of NO desorption between 300 and 400 K was attributed initially to adsorption of NO on the sample holder and on the back of the Ru (0001) substrate [94]. This weak adsorption on the stoichiometric surface is in line with a study by Yang et al. [95] that concluded that there was an adsorption of NO over the Ce^4+^ cation site on CeO_2_ (111) with a physisorbed energy amounting to only −0.1 eV. The XPS high-resolution N1s spectra showed that at 90 K, NO and N_2_O physisorbed over CeO_2_ (111). These more stable adsorbates were not reported in the study by Yang et al. [95] In two different studies conducted by Ferrizz et al. [93] and Overbury et al. [94], NO was shown to adsorb more strongly on nonstoichiometric CeO_2−y_, and these indications are in agreement with work conducted by Daturi et al. [96]. These studies linked the so-called “deNO_x_” catalytic capacity of ceria with the number of vacancies created on the ceria surface.

Ferrizz et al. [93] reported that after the NO adsorption at 300 K on nonstoichiometric CeO_2_/α-Al_2_O_3_ (0001), only N_2_ molecules were formed and desorbed. The N_2_ desorption profile is dependent on the method by which the reduced surface is synthesized. A film that was prepared in a lower O_2_ pressure led to a sharp N_2_ desorption in the range of 300 to 400 K. The desorption of N_2_ extended from 400 to 800 K by removing the O from fully oxidized films that were formed because of N_2_ desorption. Overbury et al. [94] revealed a diversity of desorbed species and showed that the desorbed product distribution was generally affected by the desorption [94]. NO, N_2_, and N_2_O molecules are formed mainly below 200 K as a consequence of adsorption at 90 K being followed by the desorption of NO and N_2_ in the range of 200 to 400 K. As the adsorption temperature increases to 150 K, the low-temperature desorption diminishes, and only additional N_2_ desorption is captured at 350 K. As the adsorption temperature rises to 400 K, new desorption characteristics appear with a considerable amount of N_2,_ in addition to the desorption of NO represented by peaks located at 500 and 740 K. A study evaluated the adsorption of the NO_2_ molecule onto ceria defective planes of (111), (110), and (100) via DFT+U approach. The adsorption of NO_2_ with the reactive sites which can perform as sites for free radical scavenging comprises an expansion in the N–O bond length as compared to that of the gas phase N–O bond length in NO_2_ [97].

## 8. Ceria Surface Reactions with CO/CO_2_ and Organic Molecules

### 8.1. Carbon Monoxide (CO)

Ceria powder and nanocrystals are known to effectively reduce CO at high temperature, forming CO_2_ and CeO_2−y_. It is very well known that the function of ceria when used in three-way catalysis is to oxidize CO gas into CO_2_. Similar to the case of H_2_, the adsorption of CO has not been reported on CeO_2_ (111) or CeO_2_ (100) under UHV conditions [98,99,100]. Many studies have reported that the adsorption of CO on ceria surfaces is very weak with a binding energy of 0.2 eV [101,102,103]. On the other hand, several studies conclude that the formation of carbonate species on the CeO_2_ (110) is an exothermic process with energy >2 eV. In another study by Nolan and Watson, the formation of carbonate was found to be more exothermic on CeO_2_ (100), −3.2 eV, than that on CeO_2_ (110), −1.95 eV [101]. Stubenrauch and Vohs [98] argued that CO could not be detected in its physisorbed state over the CeO_2_ (100) surface at 300 K. They demonstrated that 0.1 ML of CO potentially adsorbed on the CeO_2_ (100) at 100 K completely desorbed at 200 K. A projector-augmented wave (PAW) functional within generalized gradient approximation (GGA) was utilized to inspect the adsorption of CO over (111) and (110) facets. The adsorption energy on (111) and (110) surfaces was estimated to be 0.15 eV and to extended from 0.18 to 1.95 eV, respectively, which is attributed to the creation of a carbonate species [104]. A computational investigation explored CO oxidation over Pd catalyst supported over (100) and (111) directions of CeO_2_ nanocubes. The investigators illustrated that CO oxidation proceeds via a Mars–van Krevelen mechanism, which is likely to take place over the (100) facet rather than the (111) facet due to the lower Ce–O binding energy of the former [105]. A study has concluded that oxidation of CO would improve when introducing transition metals into ceria, and the catalysts follow the order of Cu > Co > Ni > Mn > Fe > Zn > pure ceria [106].

### 8.2. Carbon Dioxide (CO_2_)

Senanayake and Mullins [107] explored the weak interaction between CO_2_ and CeO_2_ (111) and found that at 90 K, the carbon dioxide was adsorbed on the surface, whilst complete desorption was recorded at 150 K. This study further reported that a minor quantity of carbonate species was produced and continued up to 300 K. In another instance, Senanayake et al. [108] demonstrated the formation of a small amount of carbonate on nonstoichiometric ceria (CeO_2−y_) grown on Au (111) substrate. These carbonate molecules persisted until 300 K. In an attempt to reoxidize the nonstoichiometric CeO_2−y_ (111) deposited on Cu (111) substrate, Lykhach et al. [109] and Staudt et al. [110] observed a negligible quantity of carbonate and carboxylate in the analyzed C 1s and O 1s curves [109]. However, a considerable concentration of Ce^3+^ was oxidized into Ce^4+^. A previous work conducted on the adsorption of CO_2_ on CeO_x_ (100) has explored a stronger interaction of CO_2_ with the oxidized and reduced surfaces [111]. In relation to the fully oxidized CeO_2_ (100), the CO_2_ was seen to be desorbed in a group of peaks at 230, 410, 510, and 655 K. The rising part of CeO_2_ (100) is indicative of CO_2_ desorption from the sample holder. The DFT+U approach was utilized to calculate and investigate the most stable sites of CO_2_ adsorption on CeO_2_ (100) and CeO_1.7_ (100). The carbonate species in tridentate form have been found to be the most stable arrangement as a result of the CO_2_ adsorption on the surface, and the adsorption energy for this species amounts to −1.93 eV [65]. It has been demonstrated that the carbonate species on the reduced CeO_1.7_ (100) is strongly stabilized at 765 K, whereas CO_2_ desorption was observed at low temperature [111]. To the best of our knowledge, there are no reports on the desorption of CO molecules on the oxidized or reduced surfaces. This is evidenced by the obtained invariant intensity of the Ce–4f peak indicating that CeO_1.7_ (100) surface has not been reoxidized by CO_2_.

The adsorption of CO_2_ on the CeO_2_ (111) face has been evaluated by Hahn et al. [112] using pure DFT and DFT+U approaches. In their simulation, they found that the most stable configuration was a monodentate carbonate species with a weak adsorption energy of nearly −0.3 eV in both approaches. Another DFT+U study conducted by Cheng et al. [113] investigated the CO_2_ adsorption on both stoichiometric and nonstoichiometric CeO_x_ (110) to reveal a physisorbed reaction between CO_2_ molecule and the surfaces with an adsorption energy of −0.26 eV. Carbonate species were not formed in their mechanism. In the last decade, CeO_2_ was used as a support for noble metal catalyst studies by Hahn [112] and Cheng [113], who reported structures of CO/CO_2_-CeO_2_ with different binding energies.

### 8.3. Hydrocarbons

When the cerium oxide surfaces are supported by Rh or Pt, ethylene adsorption and dissociation have been observed. The thermal interaction of ethylene on Rh pure crystal faces leads to the formation of gaseous H_2_ leaving C on the surface [114,115,116]. It is found that the hydrogen molecule leaves from the Rh surface between 300 and 500 K. If Rh is deposited on an unreactive material such as α-Al_2_O_3_ (0001), ethylene dissociates in an analogous way and H_2_ leaves close 440 K [117]. Ethylene dissociation over Rh/CeO_2_ (111) surface [118] or Rh/CeO_2_/α-Al_2_O_3_ (0001) forms CO and H_2_ molecules [117]. CO molecules are formed as a consequence of the reaction between the carbon layer deposited on the Rh particles and O on the ceria’s substrate. Over successive cycles of ethylene adsorption, the temperature of CO desorption shifts to increasingly higher temperatures in subsequent TPD measurements, suggesting that it becomes more difficult to eliminate O from an increasingly reduced surface [117]. Aside from CO and H_2_ production from the decomposition of ethylene on Rh/CeO_2_/yttria-stabilized zirconia (YSZ) (100), CO_2_ species were also present [119]. This indicates that the YSZ (100) substrate changes the reactivity of the O in ceria. In particular, H_2_O is not recorded in any of these Rh/CeO_2_ materials, suggesting that the H atoms produce H_2_, leaving the surface instead of interacting with O in the ceria to produce water molecules. An analogous trend is observed for ethylene adsorbed on Pt/CeO_2_ (111) catalyst [120]. The high-resolution XPS C1s spectra demonstrated the destruction of the ethylene to C on the Pt at elevated temperatures, with evidence for C atoms being desorbed. It has been shown that when CeO_2_ reduces to Ce_2_O_3_ via interaction of carbon with the surface O, eliminated carbon steadily increases. In the same vein, Pt nanoparticles on CeO_2_ (111) surfaces activate the dehydrogenation process of the ethylene at a lower temperature compared to Pt (111). Methane on Pt/CeO_2_ (111) [121,122] was shown to improve the dehydrogenation activity as well. It has been observed that methane partly dehydrogenates to CH_3_ at temperatures close to 100 K. Moreover, it has been shown that the methane species dehydrogenates to CH when reacted on Pt nanoparticles on CeO_2_ (111). Supported methane activation was also detected for low-surface-area Rh, Pt, and Pd on CeO_2_ during methane steam reforming [123].

### 8.4. Methanol

In order for organic molecules to interact with ceria surfaces under UHV conditions, the presence of a functional group including a heteroatom such as O, N, or S is essential. However, the literature presents several accounts on the interaction of a wide range of nonfunctionalized organic molecules with ceria. For instance, it has been demonstrated that ethylene leaves CeO_2_ (111) surface as a molecule under 150 K [118]. Furthermore, ethylene was investigated on Rh/CeO_2_/YSZ (100) [119], but the authors did not report ethylene’s reaction with the vacancy-free ceria surface. Instead, they focused on the interaction between the adsorbed ethylene and the Rh particles. Vile et al. studied the selective hydrogenation of alkynes to olefins at elevated conversion over polycrystalline ceria powders [124]. Carrasco et al. [125] conducted computational studies on the selective hydrogenation of acetylene to ethylene over CeO_2_ (111) using DFT. In this study, β-C_2_H_2_ radical species were reported to have adsorbed on the surface of oxygen atoms, subsequently being converted to C_2_H_3_. The formation of this species requires a lower activation energy barrier to convert to gas-phase C_2_H_4_ with reference to the formation of a β-C_2_H_4_ radical that would result in more highly hydrogenated products. Furthermore, it has been revealed that the use of lanthanide oxides as CO_2_ methanation catalysts showed an excellent catalytic activity of CO_2_ conversion of acidity from CO_2_ gas to CH_4_ gas [126]. The physisorption and chemisorption of methanol at different positions over the CeO_2_ (111) surface have been explored by DFT. The most favorable case corresponds to the dissociative adsorption of methanol via the cleavage of the C−H bond producing the coadsorbed hydroxymethyl group and hydrogen adatom. DFT results agreed with the experimental outcomes in that methanol can adsorb on a defect-free CeO_2_ (111) plane [127].

### 8.5. Phenol

Phenol plays a crucial role as a raw material in many important industrial fields such as the chemical, petrochemical, and pharmaceutical industries. Despite the positive use of phenol in the aforementioned applications, researchers have also devoted significant effort to develop methods by which phenol can be efficiently degraded owing to its well-documented role as a precursor for notorious pollutants, most notably dioxins. This is because phenol is viewed as a harmful contaminant, most notably in wastewater even at a content as low as 0.001 mg/L [128]. Furthermore, a phenolic concentration of 50 mg/L is sufficient to have a bactericidal effect on microorganisms. Lin et al. [128] investigated the catalytic efficiency of CeO_2_ on phenol conversion and total organic carbon (TOC) conversion. They used XRD, O_2_-TPD, and H_2_-TPR techniques to investigate the catalytic wet air oxidation (CWAO) of a number of CeO_2_ samples calcined under different thermal impacts, classified from the highest thermally calcined to the lowest thermally calcined, i.e., type A, type B, type C, and type D. They reported a number of conclusions. Firstly, CeO_2_ calcined under different thermal conditions displays variable catalytic efficiency in the CWAO of phenol. The highest thermal impact sample (denoted as A CeO_2_) exhibits a less stable structure, more structural oxygen exchange, and greater oxidizing capability for the intake of H_2_ and conversion of phenol. For the A-type CeO_2_, at phenol content in the range of 400 to 2500 mg/L, oxygen pressures between 0.5 and 1.0 MPa, and temperatures exceeding 160 °C, phenol conversion amounted to ratios greater than 90% after 4 h. The elimination process of total organic carbon is enhanced with the increase in the reaction temperature. Finally, CO_2_ selectivity was found to be approximately ≥80% after a 4 h reaction. Yao and coworkers [129] classified three types of oxygen in CeO_2_, namely capping, bulk, and shared oxygen. Below 500 °C, capping oxygen forms due to defects in the CeO_2_ structure undergoing an elevated thermal impact [129]. Moreover, DFT+U calculations revealed that the interaction of phenol with the hydroxylated H–OH/Ce_0.875_Mn_0.125_O_1.9375_ (111) plane is stronger than that with the clean plane [130].

## 9. Catalytic Applications

It is well known that ceria’s propensity for oxygen uptake and release, ascribed to reversible transition between Ce^3+^ and Ce^4+^, makes this material a crucial component for catalytic applications and reactions. Indeed, its catalytic activities are closely correlated to the surface structure in which different oxygen anions and cerium cations are existing on the low-index surfaces. In this section, various catalytic performances will briefly be presented.

### 9.1. Three-Way Catalysts (TWCs) in Automotive Cars

The most popular application of cerium dioxide is TWCs in which CeO_2_ or CeO_2_-based materials act as supporters to convert some harmful gases such as CO, HC, and NO_x_ emitted from automotive vehicles into more benign forms. In this process, CO and HC are oxidized to be converted into CO_2_ and H_2_O, respectively, whereas NO_x_ is reduced into N_2_. In this catalytic technology, the oxidation reactions are supported by some noble elements, namely Pt and/or Pd, whereas Rh is required to efficiently catalyze the reduction of NO_x_. Nonstoichiometric ceria (CeO_2−y_) is a good store for oxygen during lean-to-rich transients and hence plays a crucial role in further reducing NO_x_ molecules. In contrast, stoichiometric ceria (CeO_2_) is an excellent provider of the oxygen atoms needed to oxidize CO and HC during rich-to-lean transients. The TWC was initially pioneered during the 1970s–1980s, fabricated from a combination of CeO_2_ and noble metals on doped Al_2_O_3_ support. Improvements on this design were achieved in the mid-1980s by developing the CeO_2_ concentrations and optimizing the CeO_2_ distribution on the support alloyed Al_2_O_3_. Nonetheless, the formation of undesirable CeAlO_3_ and the unwanted reaction between CeO_2_ and the noble elements is significantly diminished. However, this version of TWS could not control car pollution because of poor thermal stability. The final generation of TWC convertors is the advanced TWCs that are based on CeO_2_-ZrO_2_ solid solution rather than pure CeO_2_. This version has high efficiency in removing pollutant emissions. As stated earlier, introducing ZrO_2_ into the CeO_2_ lattice enhances the oxygen storage capacity (OSC) of the system, which is needed in the redox cycles, and hence improves the efficiency of the catalyst and reduces emissions at the starting of the engine.

### 9.2. Conversion of CO_2_ to Methanol and Ethanol

One of the major hazards to nations is the emission of large quantities of carbon dioxide (CO_2_) into the atmosphere as it leads to increasing atmospheric CO_2_ concentration which in turn causes serious climate change. Thus, reusing CO_2_, instead of treating it as waste, is highly needed. In fact, a promising approach is the catalytic hydrogenation of CO_2_ to methanol. Typically, the industrial catalysts (CuO/ZnO/Al_2_O_3_) have widely been utilized according to the reaction CO_2_ + 3H_2_ → CH_3_OH + H_2_O, ΔH = −49.2 kJ/mol, in the temperature and pressure ranges of 250−300 °C and 50−100 bar, respectively [131,132]. However, this catalyst exhibits inadequate activity in converting a feed of CO_2_ and H_2_ to methanol due to the competing reverse water–gas shift (rWGS) reaction (CO_2_ + H_2_ → CO + H_2_O, ΔH = 41.2 kJ/mol). DFT+U investigation of methanol decomposing over CeO_2_ showed structure dependency in that the major product obtained depends on the facet exposed in the ceria nanostructures producing either formaldehyde or syngas as main products [133]. The high activity of CeO_2_ and CeO_2_-decorated catalysts towards CO_2_ conversion has been well documented in the literature. It has been stated that a combination of surface-modified titanium dioxide (TiO_2_) nanoparticles with reduced graphene oxide (rGO) and cerium oxide (CeO_2_) exhibited high photoreduction performances of CO_2_ conversion into methanol and ethanol fuels by yielding methanol and ethanol at 641 and 271 μmol g^−1^ h^−1^, respectively [134]. Moreover, CO_2_ hydrogenation activity demonstrated that the Cu/CeO_2_ catalysts had higher methanol selectivity compared to Cu/SiO_2_ catalyst. The superior methanol selectivity is attributed to the inhibition of the rWGS activity [135]. The hydrogenation of CO_2_ to methanol over Au/CeO_2_ catalysts has been reported [136]. Results demonstrate that CO hydrogenation to methanol over Au/CeO_2_ is hindered by the presence of CO_2_, indicating that the carbonate-like species can block the active sites at the Au-CeO_2_ interface. Abdullah et al. [137] investigated the performance of a CeO_2_-TiO_2_ photocatalyst for the photocatalytic reduction of CO_2_ into methanol under visible light irradiation, and their results suggested that the CeO_2_-TiO_2_ exhibited superior photocatalytic performance by producing methanol at 18.6 µmol/g under visible light irradiation when compared with the bare TiO_2_ (6.0 µmol/g).

### 9.3. Oxidation of Volatile Organic Compounds (VOCs)

Chlorinated volatile organic compounds (CVOCs) are a group of widespread contaminants that are emitted as gases that may lead to short- and long-term health problems. Therefore, the elimination of these compounds that have been widely detected indoors and outdoors has gained considerable attention [138]. CeO_2_ and CeO_2_-based materials have been employed as economic alternatives to RuO_2_-based catalysts to oxidize and decompose HCl, a harmful by-product produced by industrial processes such as polycarbonate production from dehydroxylated organics and organic chlorination reactions. The chlorine molecule Cl_2_ is found to be the predominant industrial output for HCl removal [139]. CeO_2_-based catalysts were noted to be active in O_2_-rich feeds (O_2_/HCl ˃ 0.75), whereas the deactivation of the catalysts was explored in O_2_-poor feeds (O_2_/HCl ˂ 0.25). High Cl coverage hinders the formation of oxygen vacancies. Hence in order for the original activity to be restored, the samples should be exposed to an excess of oxygen, indicating a reversible deactivation due to the chlorination [140]. Furthermore, CeO_2_-ZrO_2_ solid solution as an improved catalyst exhibits prolonged stability (700 h on stream) and lessens the chlorine uptake compared to undoped CeO_2_ [141].

**Figure 9:**
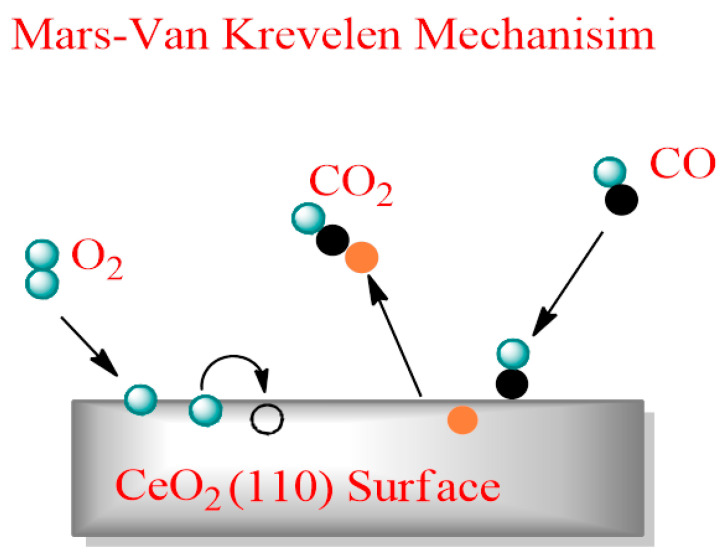
Schematic illustration of the CO oxidation over CeO_2_ (110) surface via Mars‒van Krevelen mechanism [148].

Various pathways for VOC treatments have been reported, including adsorption, direct combustion, regenerative combustion, and catalytic combustion [142]. CeO_2_ and CeO_2_-based compounds have served as oxidizing agents for the treatment of VOCs. The use of a catalyst reduces the activation energy and makes it possible to oxidize VOCs at a lower temperature. The catalytic oxidation of benzene (B), chlorobenzene (CB), and 1,2-dichlorobenzene (1,2-DCB) over W-modified Pt/CeO_2_ catalysts has been studied [143]. A decrease in the strength of Cl adsorption on Ce and Pt sites can be promoted by the addition of W. The interaction of W with Pt species led to the formation of Pt–O–W structures, which promoted the reducibility and availability of surface oxygen. For controlling the emission of VOCs in industries such as painting, printing, and gluing, Pt/Co_3_O_4_-CeO_2_ catalyst was effective in lowering the oxidation temperature of toluene, ethyl acetate, and isopropyl alcohol at less than 250 °C [144]. A Mars–van Krevelen-type mechanism governs the reaction in which CeO_2_ acts as the oxygen provider and at the same time is reoxidized by the gas-phase oxygen [145,146]. High-surface-area CeO_2_ materials have been well known as promoters for noble metals (Pd, Pt, and Au). CeO_2_ is now being deployed as a low-temperature catalyst in the decomposition of VOCs. This is because of the increase in metal dispersion and CeO_2_ participation in the reaction [147]. Figure 9 illustrates the Mars–van Krevelen mechanism of the CeO_2_ (110) surface towards oxidizing CO. This mechanism commences with the adsorption of CO and implicates the contribution of an oxygen atom resulting in the formation of desorbed CO_2_ and an oxygen vacant site. Next, the oxygen vacancy is wrapped by O_2_ that reoxidizes the surface.

Methane (CH_4_), as one of VOCs, is known to pose a number of issues such as its potential impact on global warming and its major contribution to ozone depletion [149]. The combustible nature of CH_4_ adds further challenges in any process aimed at tackling its removal/conversion. The catalytic efficiency of a CeO_2_-ZrO_2_ solid solution synthesized by urea hydrolysis toward methane was demonstrated to depend on the Ce:Zr fraction. The most active composition was observed in Ce_0.75_Zr_0.25_O_2_, and a gradual drop in the activity was recorded when Zr content decreased due to the phase change and modification of redox properties. Although these systems have been recognized to possess high thermal stability traits, they suffer from a general catalytic deactivation during light-off experiments (i.e., the catalyst light-off is the minimum temperature necessary to initiate the catalytic reaction). By a more precise definition, the light-off temperature is the temperature at which conversion reaches 50%. Liotta and coworkers suggested that the strong electron transfer between Ce_3_O_4_ and CeO_2_ resulted in enhanced redox properties and improved methane oxidation.

This enhancement has also been observed with CuO/CeO_2_ catalysts when optimizing the CuO dispersion, the metal loading, and the electronic interaction with ceria, resulting ultimately in the improved catalytic activity of the system. Despite the excellent efficiency of the CuO/CeO_2_ solid solution, a noticeable decrease in the activity was observed due to the existence of H_2_O. In a Ce_0.9−x_Cu_0.1_Ca_x_O_2−y_ system, a remarkable improvement was observed following the introduction of Ca as a result of the formation of oxygen vacancies. This system losses its activity over time due to the migration of Ca atoms to the surface, thereafter forming calcium and carbonate species. Finally, the incorporation of La in La_x_Ce_1−x_O_2−y/2_ solid solutions led to the enhanced reducibility of ceria and, at the same time, an increase in the formation of oxygen vacancies and surface superoxide ions. In fact, these solid solutions were found to be crystalized in very small sizes as the ratio of Ce/(Ce + La) was kept in the range from 1.0 to 0.2.

The addition of noble metals such as Pt to different compositions of Ce_x_Zr_1−x_O_2_ catalysts has been previously found to produce thermally stable structures. Among the investigated systems, Pt/Ce_0.67_Zr_0.33_O_2_ was found to be the most thermally stable and the best active catalyst at 1000 °C. Pt/Ce_0.67_Zr_0.33_O_2_ catalysts were also found to be much more active than Pt/Al_2_O_3_. Several important catalyst systems, along with a summary of their synthesis and other operational information (BET surface area, gas hourly space velocity (GHSV), and temperature), are tabulated in Table 3.

Ce_1−x_Zr_x_O_2_ (x = 0–0.3) exhibits a very good catalytic efficiency towards the oxidation of some non-methane gases, especially benzene and toluene. The Ce_1−x_Zr_x_O_2_ solid solution has been proven to be more efficient than unalloyed CeO_2_ in the combustion of these harmful molecules. For instance, the Ce_0.9_Zr_0.1_O_2_ solid solution demonstrated higher catalytic performance toward oxidizing benzene and toluene than pure CeO_2_ at a temperature of 100 °C lower than light-off temperature, the temperature at which 50% of conversion (T_50_) is completed [163]. In a similar context, MnO_x_-CeO_2_ mixed oxides exhibit excellent catalytic performance for formaldehyde combustion. The synthesis method of such a mixed oxide is an important factor in tuning its catalytic performance. The improvement is due to the formation of a higher oxidation state of manganese and more oxygen on the surface resulting in an enhancement in the energy barrier for the oxygen transfer mechanism [159].

### 9.4. Decomposition of Chlorinated Volatile Organic Compounds (CVOCs)

As stated previously, CVOCs are toxic materials that are emitted from industrial waste gases and contribute significantly to air pollution nowadays. These compounds are emitted from thermal processes whenever a trace of chlorine coexists with hydrocarbon entities. Among these pollutants are the notorious polychlorinated dibenzo-p-dioxins and dibenzofurans [164,165]. As a result, significant attention has been devoted to controlling the emission of these pollutants via the development of novel and efficient catalysts. Catalytic oxidative decomposition is currently deployed as an alternative for the commonly deployed high-temperature operations. Initially, noble metal (e.g., Pt and Pd) catalysts or supported noble metal catalysts were extensively employed to decompose VOCs [166,167,168]. These catalysts are very active in the catalytic destruction of chlorinated VOCs; however, they can be readily poisoned by emitted HCl and Cl_2_ gases [169]. Transition metal oxide catalysts have now emerged as a cost-effective alternative candidate to noble metals to carry out deep catalytic oxidation for CVOCs. Although they are somewhat less active than noble metals, they are preferred because of their low price and resistance to HCl/Cl_2_ poisoning [170]. The catalytic capacity of CeO_2_ in acting as a stand-alone environmental catalyst toward the decomposition of a series of chlorinated volatile organic compounds, namely chloroethene, chloroethane, and chlorobenzene, has been highlighted via DFT approach [171]. The pyrolytic and oxidative decomposition of selected chlorinated compounds has been modeled on the most stable ceria surface, CeO_2_ (111). The findings revealed that the separation of the C-Cl bond over oxygen vacancies systematically necessitates lower energy barriers in reference to clean surfaces. CeO_2_ has been examined for the catalytic combustion of trichloroethylene (TCE), and Dai et al. investigated the catalytic performance in eliminating some chlorinated VOCs. They concluded that CeO_2_ demonstrates an effective catalytic capacity in decomposing Cl-VOCs at low operating temperatures. They also found that the catalytic removal of chloroalkanes over the CeO_2_ catalyst is easier than that of chlorinated allylenes [172]. Dia et al. [169] studied trichloroethylene combustion in a broad range of reaction temperatures over CeO_2_ systems. Dia et al. attribute the lower efficiency of the CeO_2_ calcined at 650 and 800 °C, in reference to that at 550 °C, to a diminution in BET surface area and an increase in crystallite size. The lower catalytic efficiency for CeO_2_ calcined at 450 °C is attributed to it having fewer basic sites and active oxygen species.

### 9.5. Full Hydrogenation of Ethyne

**Figure 10:**
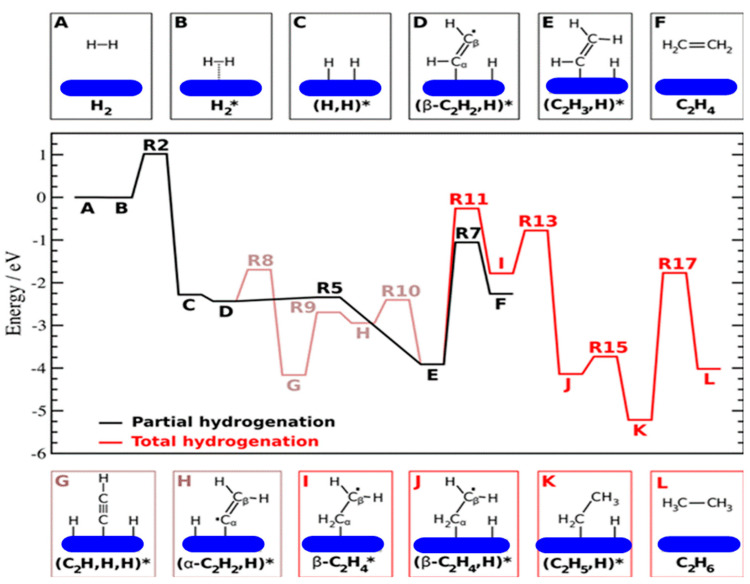
Reaction energy diagram for total hydrogenation of acetylene on CeO_2_ (111) catalyst. Energies are computed as a reference to the energy of H_2_ and C_2_H_2_ in the gas phase and the clean CeO_2_ (111) slab. Black line shows the routes of partial hydrogenation of acetylene to ethylene through R5, light brown displays the partial hydrogenation via dissociative acetylene adsorption, and red line represents the full hydrogenation to C_2_H_6_. The asterisk denotes a clean CeO_2_ (111) surface. Reactants, intermediates, and products that are followed by an asterisk correspond to the adsorbed species. The asterisk in reactants, products, and intermediates correspond to adsorbed moieties [125].

The overall hydrogenation process of acetylene (C_2_H_2_) to ethane (C_2_H_6_) has been determined experimentally. A plausible hydrogenation mechanism was suggested based on DFT calculations. The entire energy landscape and primary reaction steps along with all intermediate configurations are depicted in Figure 10. It has been suggested that geometry optimizations for the adsorption of each of the investigated species preferentially occur over the surface O atoms. Hydrogen molecule adsorption and its subsequent dissociative adsorption on the surface are supposed to be the starting steps rather than the adsorption of a gas-phase C_2_H_2_ molecule. As a result, the reaction is initiated with the molecular adsorption of H_2_ followed by homolytic dissociative adsorption to leave two H* species and liberate 2.35 eV. The two H atoms adsorb over two closest neighboring surface O atoms to produce two hydroxyl groups and two Ce^3+^. This primary step necessitates an activation energy of 1.00 eV. The step to separate the two closest adjacent H* species resulting in two separated species, (H, H)* → H* + H*, is energetically unfavorable by 0.07 eV. The reaction continues with the adsorption of an acetylene molecule close to one hydroxyl group to form (β-C_2_H_2_, H)* (step D in Figure 9). Subsequently, the radical can easily react with the adjacent hydroxyl groups to produce C_2_H_3_* and liberate 1.48 eV. Notably, this process involves only a very small energy barrier of 0.09 eV. In the subsequent step, an adsorbed hydrogen atom migrates to the radical center in the C_2_H_3_*, forming an ethene molecule. The fate of the ethene molecule is dictated by two competing channels, desorption into the gas phase or subsequent two hydrogenation steps toward the formation of an ethane molecule, i.e., full hydrogenation route. It has also been reported that various U values would influence the reaction and activation energies for the full hydrogenation reaction of C_2_H_2_ to C_2_H_6_ over (111) CeO_2_ surface catalyst [173].

### 9.6. Soot Combustion

This context highlights the recent advances in preparing various shape-controlled CeO_2_ particles and examining their catalytic performances in soot combustion. Nanofibers of CeO_2_ were synthesized aiming at improving the soot–catalyst contact conditions and promoting soot combustion at lower temperatures than in the noncatalytic case. In particular, the nanofibers have been found to be very active with respect to other ceria catalyst morphologies due to their arrangement in a network that enhances the number of soot–fiber contact points [174]. Aneggi et al. [175] fabricated two types of shape-controlled nanoceria in the forms of cubes and rods by hydrothermal methods then examined their catalytic performance for soot combustion. Results showed that soot oxidation activity and conversion are higher for nanocubes and nanorods compared with conventional polycrystalline ceria and are affected by the nature of the exposed facets. Zhang et al. [176] synthesized three sorts of morphologies, namely nanorod, nanoparticle, and nanoflake, and examined the relation between the catalytic performance of ceria for soot combustion and the shapes. The findings indicated that CeO_2_ with nanorod morphology revealed the best catalytic activity, and it reached a level comparable to that of a precious metal catalyst.

## 10. Applications

### 10.1. Photocatalytic Performance

Worldwide, efficient visible-light photocatalysis is an active area of research. Photocatalytic activity with the application of sunlight offers an inexpensive and green technology for possible complete removal of refractory pollutants that damage the ecosystem, such as surfactants, pharmaceuticals, pesticides, textile dyes, and heavy metals, from industrial wastewater [177]. Literature reported the use of CeO_2_ in photocatalytic applications, whether it is loaded, doped with metal species, or coupled with other materials. This use is supported by high bandgap energy, high refractive index [178], high optical transparency in the visible region [179], and excellent oxygen storage capacity resulting from the easy conversion of cerium ions between reduced and oxidized states (Ce^3+^–Ce^4+^) through fast creation and removal of oxygen vacancies in CeO_2_. This section reports the recent photocatalysis-based studies of pure and doped CeO_2_. The photocatalytic activity of CeO_2_ is mainly related to particle size, surface structure, and morphology. An effective catalyst for the photodegradation of crystal violet dye under UV light has been fabricated based on noble metal oxide catalysts. Majumder et al. [180] tailored various crystallographic structures (hexagonal, rectangular, and square) and planes including (100), (111), and (101) of CeO_2_ nanostructures and tested their photodegradation activities on methylene blue (MB) dye. The bandgap is reduced up to 1.93 eV and the complete degradation of methylene blue is achieved within 175 min, which is the shortest to be reported as compared with the complete degradation time of MB that is reduced from 23 to 14 h using samarium- and gadolinium-doped CeO_2_ nanoparticles instead of bare ceria. A study was carried out to evaluate the role of various contents of Sn^4+^ incorporated into CeO_2_-Fe_2_O_3_ nanocomposites in the degradation of MB and methyl orange (MO) dyes under visible light. They found that the catalytic activity was improved due to the shifting of the Fermi level of CeO_2_ by narrowing the bandgap energy (~2.3 eV) and enabling charge separation. Sm-doped CeO_2_ nanorods were annealed in a N_2_ atmosphere to obtain defective Sm-doped CeO_2_ photocatalysts that demonstrated excellent performance in the photodegradation of MB under visible light irradiation [181]. Additionally, a DFT outlook showed that inserting Ti into the ceria lattice has an important influence on lowering the bandgap of the undoped system, which shows promise in the fields of photocatalysts [182]. Furthermore, the effect of Cr doping on CeO_2_ nanostructure was investigated to assess the catalytic performances of Cr-doped CeO_2_ nanocomposites with MB. They observed an improvement in the catalytic activity in MB degradation efficiency for photocatalysis [183]. In another study, CeO_2_ nanoparticles were decorated on a graphene sponge and utilized for adsorption and photodegradation of methylene blue [184].

### 10.2. The Biomedical Applications

Nanoceria has substantial applications in the biomedical field, including orthopedic biomedicine and specifically bone tissue engineering, which have widely utilized ceria nanoparticles [185,186]. A wide array of biomedical applications have been covered in the literature [187]. In a recently published study, near-spherical cerium oxide nanoparticles (CeNPs) stabilized by various hydrophilic polymers were synthesized through a wet-chemical precipitation method to inspect the impacts of CeNP surface properties on their colloidal stability and catalytic activity in human cellular media. Two types of forces generated by surface functionalization have been deployed to stabilize the CeNPs: electrostatic forces, steric forces, or a combination of both. To enhance CeNP colloidal stability, polyacrylic acid (PAA) has been used as a good coating agent. Results indicate that the most stable sample in cell culture media was PAA-coated CeNPs with a combination of both electrostatic and steric forces on the surface. The sample also showed nontoxicity toward the osteoblastic cells and displayed promising biomedical applications [188]. Miri et al. [189] reported on the biosynthesis of CeO_2_ nanoparticles with an estimated particle size ranging from 10 to 15 nm. The synthesized nanoparticles revealed a nontoxic effect against a colon cancer cell line. Moreover, nanoceria has demonstrated free radical scavenging activity and the ability to offer protection against ionizing radiation [190]. Besides, the inadequate radiopacity of dental adhesives applied under composite restorations makes the radiographic diagnosis of recurrent caries challenging. Therefore, the misdiagnosis leads to the unneeded replacement of restorations. To solve this issue, incorporating elements with a high atomic weight can enhance the radiopacity of dental adhesives. This motivated researchers to employ CeO_2_ particles to enhance the radiopacity of the dental adhesives [191].

## 11. The Effect of Facets on the Catalytic Applications of CeO_2_

The morphology- and surface-dependent catalytic activity of CeO_2_ nanomaterials has drawn much attention in the literature, and findings suggest that the shape of CeO_2_ crystals is mainly related to defect sites of the well-defined crystal planes. Thus, the correlation between catalytic properties and specific morphology (in which different crystal planes are exposed) can be achieved via exposure of different surfaces in addition to the most stable (111)-type plane of the nanocrystal [192]. The adjusted morphologies of CeO_2_ as particles, spheres, cubes, rods, wires, and tubes with specific directions (111), (110), and (100) can remarkably improve catalytic performances. It has been stated that designing heterogeneous junctions is an attractive approach for refining the catalytic properties of CeO_2_*-*based nanomaterials. Moreover, modifying defective structures by inserting minor Mn contents as dopants can improve the catalytic activity. Thus, hydrothermally fabricated CeO_2_ nanowires/MnO_2_ nanosheets showed improved catalytic performance because of the strong synergistic interaction between CeO_2_ and MnO_2_ at the interface [193]. Two shapes of controlled nanoceria, namely spherical and cubical, were synthesized through the hydrothermal method and by tuning reaction temperatures. The cubical NPs demonstrated superior degradation of ~70% under UV light irradiation compared to the spherical ones (degradation of <50%). The antibacterial activities of both NPs were also examined, and cubical NPs were found to exhibit superior antimicrobial potential. This implies that cubical NPs demonstrate a propensity for treating industrial wastewater and inhibiting the growth of different microorganisms which in turn be useful in developing medical devices and tailoring various antimicrobial systems [194].

Dong et al. [195] used hydrothermal and solvothermal methods to synthesize a series of Cu/CeO_2_ catalysts with diverse morphology and size, comprising CeO_2_ nanoparticles (20 nm), CeO_2_ nanospheres (200 nm), CeO_2_ nanorods (20–40 nm), and flower-like CeO_2_ microspheres (4 µm). The prepared catalysts were tested for the catalytic oxidation of CO under dry and humid conditions to explore the shape effect on CO oxidation performance. Cu/CeO_2_ nanorods demonstrated the smaller particles size of CeO_2_ and CuO and displayed the higher concentration of oxygen vacancies. Moreover, the best catalytic performance of about 90% conversion of CO at the temperature of 58 °C was detected. Li et al. [196] used the hydrothermal method to fabricate ruthenium (Ru) catalysts on various shapes of supported CeO_2_, including nanorods, nanocubes, and nano-octahedra. Ru supported on CeO_2_ nanorods revealed boosted low-temperature hydrogen consumption and superior room-temperature CO oxidation activity of <100 °C and ∼9% CO conversion, respectively. Lastly, the surface morphologies of CoO_x_-decorated CeO_2_ heterostructured catalysts, including nanorods (NRs), nanocubes (NCs), and nanoparticles (NPs), were found to strongly correlate with the physicochemical properties and catalytic performance toward diesel carbon soot oxidation. The CoCeO_2_-NRs boosted soot combustion activity and stability at lower temperatures compared to CoCeO_2_-NCs and CoCeO_2_-NPs. This result is mainly attributed to the high oxygen release rate and improved redox capability of the supported Co species. This originates from the high reactivity of oxygen atoms on (110) surfaces compared to (100) and (111) surfaces over CeO_2_ [197]. Finally and from another prospect, it has been stated that the catalytic properties of CdS can be enhanced by rare earth doping [198].

## 12. Conclusions

In this review, we summarized the fundamental aspects pertinent to catalytic properties, applications, morphologies, and structures of ceria. Due to the remarkable properties such as high surface area, superior electronic properties, and outstanding optical properties, ceria has widely been utilized in electronics and organic synthesis reactions. On the other hand, distinctive redox properties and defect-rich geometry enable ceria to exhibit excellent catalytic performance in various reactions. CeO_2_ (ceria) can catalyze a wide range of reactions by assisting oxidation and reduction and hydrogenation and dehydrogenation. Besides, ceria can be active as a crystal or as a polycrystalline layer supported by a metal or metal oxide, commonly Al_2_O_3._ The present review also highlights the recent applications of ceria in electronic components and nanomedicine. Defective ceria shows enhanced catalytic activity, and solid solutions (e.g., ceria with zirconia) display enhanced catalytic activity. Furthermore, zirconia-doped ceria (i.e., Ce_x_Zr_1−x_O_2_) makes an effective catalyst when x is optimized. Achieving desired morphologies with tailored crystal facets and oxygen vacancy in ceria through *a* controlled synthesis process has been reported, as this is necessary for highly active/selective heterogeneous catalysts. In addition, ceria nanostructures are more effective than polycrystalline ceria. Finally, this account involved computational modeling investigations highlighting the effects of ceria crystal facets and morphology on the desired properties and applications. We envisage that future research can focus on several aspects, most notably the following:

- The effect of new dopants such as Nb and Ta should be assessed in deriving the capacity of ceria in low-temperature oxidation.
- Molten metals have been recently deployed as heat transfer media and catalytic reagents. It would be insightful to assess the influence of Ce atoms in the emerging energy application of liquid metals.
- In an analogy to the role of ceria in dehalogenation reactions, it would be insightful to explore the potential role of ceria in fixing the fluorine content in C_n_F_m_ chemicals.

## Figures and Tables

**Table 2:** Structural changes of stoichiometric and nonstoichiometric CeO_2_ with temperature.

Material	Oxidation Extent, *y*	Temperature (°C)	Thermal Treatment	Structural Phase
**CeO_2−*y*_**	0	˂685	–	Fluorite structure (*fcc*)
**CeO_2−*y*_**	0 ˂ *y* ˂ 0.286	˃685	–	*α* phase (disordered fluorite structure)
**CeO_2−*y*_**	0.166	˃685	Thermally treated	*β* phase (ordered fluorite, monoclinic structure)
**CeO_2−*y*_**	0.181	˃685	Thermally treated	*δ* phase (triclinic structure)
**CeO_2−*y*_**	0.285	˃685	Thermally treated	Rhombohedral structure
**CeO_2−*y*_**	˃0.286	˃685	–	*σ* phase (*C*-type Ce_2_O_3_, *bcc*)

**Table 3:** Summary of the most important catalysts used for catalytic combustion of VOCs.

Catalyst	Synthesis Technique	BET Surface Area (m^2^ g^−^^1^)	VOC	GHSV (mL g^−^^1^ h^−^^1^)	VOC Concentration	T_50_^b^ (°C)	Ref.
**Ce_0.75_Zr_0.25_O_2_**	sol–gel	108.4	methane	60,000	2%	545	[150]
**5 wt% Cu/CeO_2_**	hydrothermal	22.6	methane	27,000	1%	540	[95]
**1 wt% Cu/CeO_2_**	thermal decomposition	68.7	methane	54,000	1%	540	[95]
**Ce_0.85_Cu_0.1_Ca_0.05_O_2−δ_**	citric acid complexation combustion	31.3	methane	30,000	1%	478	[151]
**Ce(0.6)-La-O**	sol–gel	52.4	methane	13,500	0.2%	505	[152]
**Co_3_O_4_-CeO_2_**	coprecipitation	31	methane	60,000	0.3%	471	[153]
**2 wt% Pt/Ce_0.67_Zr_0:33_O_2_**	impregnation	79	methane	12,800	1%	550	[154]
**CeO_2_**	sol–gel	3	toluene	200,000	1000 ppm	430	[147]
**5 wt% CeO_2_/Al_2_O_3_**	impregnation	156	toluene	54,000	1400 ppm	275	[155]
**Ce_0.9_Zr_0.1_O_2_**	sol–gel	56	toluene	20,000	1000 ppm	221	[156]
**Ce_0.9_Zr_0.1_O_2_**	sol–gel	56	ethanol	20,000	1000 ppm	207	[156]
**CuO-CeO_2_/γ-Al_2_O_3_**	impregnation	156	propane	2300	5.9%	350	[157]
**Cu_0.13_Ce_0.87_O_2_**	combustion	27	acetone	60,000	1000 ppm	200	[158]
**MnOx-CeO_2_**	sol–gel	22.2	formaldehyde	60,000	580 ppm	160	[159]
**MnO_x_-CeO_2_**	modified coprecipitation	124	benzene	30,000	200 ppm	260	[159]
**3 wt% Ag/MnO*_x_*-CeO_2_**	deposition precipitation	124.0	formaldehyde	30,000	580 ppm	70	[160]
**0.5 wt% Pt/CeO_2_**	impregnation	3	toluene	200,000	1000 ppm	180	[147]
**1.5 wt% Au/CeO_2_**	deposition precipitation	79	propene	35,000	1000 ppm	230	[161]
**0.25 wt% Pt/23wt% CeO_2_/Al_2_O_3_**	sol–gel	95	acetic acid	30,000	1000 ppm	175	[162]
